# Bone Regeneration and Oxidative Stress: An Updated Overview

**DOI:** 10.3390/antiox11020318

**Published:** 2022-02-06

**Authors:** Adrian Emilian Bădilă, Dragos Mihai Rădulescu, Andrei Ilie, Adelina-Gabriela Niculescu, Alexandru Mihai Grumezescu, Adrian Radu Rădulescu

**Affiliations:** 1 “Carol Davila” University of Medicine and Pharmacy, 050474 Bucharest, Romania; adrian.badila@umfcd.ro (A.E.B.); dragos.radulescu@umfcd.ro (D.M.R.); radu_radulescu@umfcd.ro (A.R.R.); 2 Department of Orthopedics and Traumatology, Bucharest University Hospital, 050098 Bucharest, Romania; 3 Faculty of Engineering in Foreign Languages, University Politehnica of Bucharest, 011061 Bucharest, Romania; andrei.ilie.97@gmail.com; 4 Faculty of Applied Chemistry and Materials Science, University Politehnica of Bucharest, 060042 Bucharest, Romania; adelina.niculescu@upb.ro; 5 Research Institute of the University of Bucharest—ICUB, University of Bucharest, 050657 Bucharest, Romania; 6 Academy of Romanian Scientists, 3 Ilfov Street, 050044 Bucharest, Romania

**Keywords:** bone formation, BTE, mesenchymal stem cells, osteoclasts, ROS, oxidative stress

## Abstract

Bone tissue engineering is a complex domain that requires further investigation and benefits from data obtained over past decades. The models are increasing in complexity as they reveal new data from co-culturing and microfluidics applications. The in vitro models now focus on the 3D medium co-culturing of osteoblasts, osteoclasts, and osteocytes utilizing collagen for separation; this type of research allows for controlled medium and in-depth data analysis. Oxidative stress takes a toll on the domain, being beneficial as well as destructive. Reactive oxygen species (ROS) are molecules that influence the differentiation of osteoclasts, but over time their increasing presence can affect patients and aid the appearance of diseases such as osteoporosis. Oxidative stress can be limited by using antioxidants such as vitamin K and N-acetyl cysteine (NAC). Scaffolds and biocompatible coatings such as hydroxyapatite and bioactive glass are required to isolate the implant, protect the zone from the metallic, ionic exchange, and enhance the bone regeneration by mimicking the composition and structure of the body, thus enhancing cell proliferation. The materials can be further functionalized with growth factors that create a better response and higher chances of success for clinical use. This review highlights the vast majority of newly obtained information regarding bone tissue engineering, such as new co-culturing models, implant coatings, scaffolds, biomolecules, and the techniques utilized to obtain them.

## 1. Introduction

There are many circumstances in which bone defects can occur, such as trauma, congenital origins, or disease, impacting millions worldwide [1,2]. The defects present a great challenge for surgeons in cases of sizable osseous defects [3]. The lacunes existing in this area determined the necessity of comprehending the process of bone regeneration in order to generate better solutions [4].

Bone tissue is intensely vascularized, a particularity that influences growth, maturation, shaping, and regeneration [5,6]. Bone tissue can be of two types: cortical bone, which represents the external part of a bone, and cancellous bone, which is located inside the bone. The differences between these two layers are rigidity and porosity. The cortical bone is less porous and presents better mechanical stiffness than cancellous bone, which has approximately ten percent of the cortical bone stiffness [7,8,9]. Osteons inside the cortical bone form units with a Haversian system containing nerves and blood vessels [10]. Cancellous bones do not possess osteons with the Haversian system, but this aspect does not impact the blood vessel stream, which is a consequence of the high bone porosity. In the embryonic stage, bone can form in two distinct methods of ossification: intramembranous and enchondral [11].

Oxidative metabolism produces the reactive oxygen species (ROS) as a byproduct of energy-generating reactions that are largely generated in the mitochondria. A beneficial way in which reduced levels of ROS can operate is as signaling molecules that are vital for balancing cell differentiation, self-renewability, and proliferation. On the other hand, raised levels of ROS are damaging due to the interaction frequency with molecules such as proteins, RNA, and DNA, thus resulting in osteogenic lineage suppression [12]. One crucial factor in bone regeneration is maintaining bone homeostasis. In normal bone homeostasis, osteoblasts differentiation utilizing signal pathways such as fibroblast growth factor (FGF), bone morphogenetic protein, and hedgehog is facilitated; at the same time, the differentiation of osteoclasts is modulated with the aid of macrophage colony-stimulating factor (M-CSF) and the receptor activator of nuclear factor kappa-B ligand (RANKL) [13]. 

Therefore, this review aims to present the main tools of bone tissue engineering, precisely the scaffolds such as hydroxyapatite and bioactive glass, and the enhancing agents such as growth factors and biomolecules. Furthermore, the models of co-culturing and other studies concerning oxidative stress and reactive species of oxygen have been focused on to observe osteoclasts, osteocytes, and osteoblasts’ influence in the domain, both in single-cell form and together.

## 2. Bone Regeneration

Several methods address and enhance the regeneration of damaged tissue, which aim to bypass the limitations encountered by already utilized treatments, such as functionalization issues, lack of material compatibility to certain techniques, and translational success limitation [14,15]. Biomaterials and scaffolds can promote healthy tissue formation and are sought in fields such as bone regeneration [16]. Successful approaches orient towards materials that present a good biomimetic property and are bioactive to obtain similar structural features compared to the original extracellular matrix (ECM) [17,18,19]. 

Hydrogels are a great example when it comes to materials that influence the bone tissue engineering (BTE) field by releasing different types of growth factors (GFs) that aid neovascularization. There are three types of hydrogels that include various options: natural [16], semi-synthetic, and synthetic [6,20,21,22].

**Figure 1:**
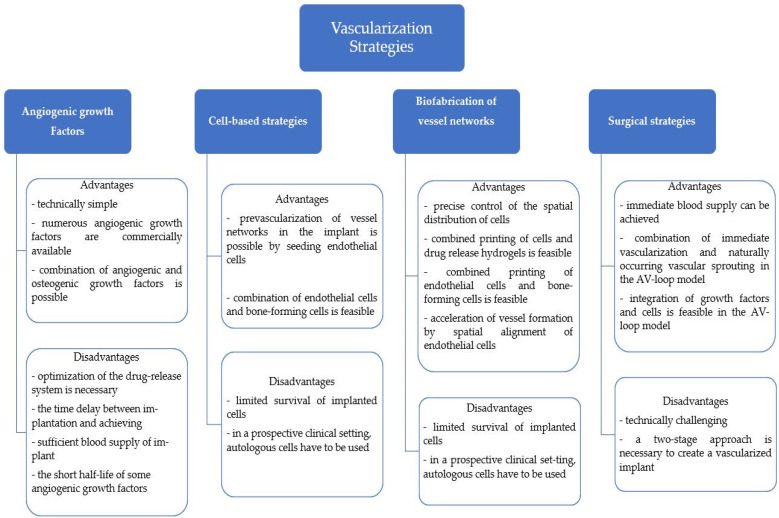
Advantages and disadvantages of vascularization strategies. Created based on information from [7].

A class of materials that are commonly used for bone regeneration applications are bioceramics such as calcium sulfate (CS), hydroxyapatite (HA), and calcium silicate, which are frequently utilized because of their low cytotoxicity and high bioactivity and biocompatibility [23,24]. Bioceramic materials possess a microstructure that promotes ossification and vascularization growth, vital characteristics for osteointegration and osteoinduction [25,26]. This wide class of materials can be utilized as scaffolds which are characterized by properties such as pore shape and size, porosity, crystal distribution, sinter temperature, and phase composition [27,28,29]. Figure 1 presents the advantages and disadvantages of a selected array of vascularization strategies.

## 3. Hydroxyapatite for BTE

**Figure 2:**
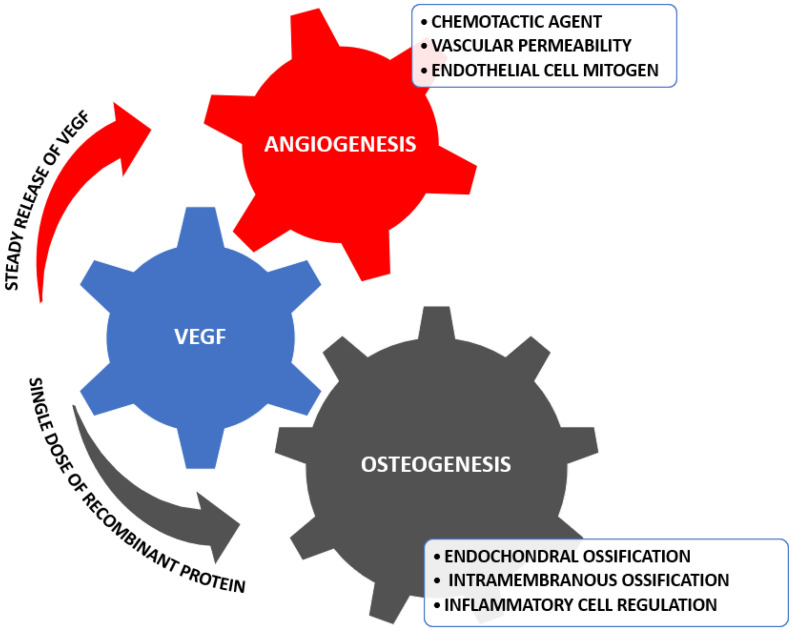
Regulations occurring from VEGF signal. Created based on information from [36,37].

Among the strategies developed in the domain of regenerative medicine that strive to duplicate the tissue in order to obtain an efficient result, we can count materials such as bivalent aptamer-conjugated hydroxyapatite (Apt-HA) [30,31]. Apt-HA’s particularity is the specifically adsorbed vascular endothelial growth factor (VEGF), and it is utilized in synergetic regeneration and osteoconduction [32,33]. The functionalization with growth factors has been discovered recently, and it is realized by immersion; this led to a growth in research for the bioactivity enhancement of bioceramic scaffolds [34,35]. VEGF is one of the first proteins that presented links to osteogenesis and angiogenesis, the inactivation process being an instrument of observation for bone formation and vascularization [36,37]. VEGF and its receptors are expressed by osteoclasts, osteoprogenitors, and osteoblasts, and signal by promoting differentiation, activity, and recruitment [38]. Figure 2 displays the regulation that VEGF realizes to stimulate angiogenesis and osteogenesis.

There are multiple variations in which HA can be obtained depending on the source and quantities of the precursors. A suitable type for BTE can be carbonated hydroxyapatite (CHA), which serves as a coating on metallic substrates, preventing them from releasing metallic ions and protecting them from corrosion [39,40]. The material development is oriented towards mimetism, which guides parameters such as crystallinity, microstructure, and chemical composition. Mineral carbonate (CO_3_)^2−^ is a component of natural bone that is present in a range of 2%–8% [41]. CHA is obtained by enriching the HA with carbonate minerals such as natural bone [42]. Several sources of bio-waste, such as eggshells, seashells, and animal bones, are rich in calcium, being suitable candidates for medical-grade sources of obtaining HA [43,44,45]. Several technological fluxes are available for obtaining a diversity of CHA with different properties, including co-precipitation [46] sol-gel, nanoemulsion [47], mechanochemical-hydrothermal [48], and mechanical alloying. The most sought method is co-precipitation due to the reduced costs and large-scale production capacity [49].

Another beneficial aspect of HA is the buffering mechanism ensured by PO_4_^3−^ and OH^−^; the ions of calcium and phosphate aid the remineralization, opposing the effect of calcium carbonate [50,51,52,53]. HA can influence the pH of the cariogenic biofilm, increasing it from 4.3 to 4.8. Apart from their use for mineralization enhancement, HA and CHA can also be used as buffering agents for organic acids [54,55].

Osteoporosis, which can be considered a discrepancy between osteoclasts-related bone resorption and osteoblasts-related bone development, can be caused by an estrogen deficiency [56,57]. The condition results in the mass reduction of cortical and trabecular bone, causing skeletal weakness and fractures. A suitable material for this application is nano HA (nHA), generating signals that stimulate the cell desired behavior and bone biomarker activity [58]. nHA is a great option due to the influence of the particle size on the strength of the implant coating; the scaffold should sustain bone deposition and the forces that are exerted [59].

## 4. Bioactive Glass

Silicate-based bioactive glass (Si-BaG) has become a very popular material for clinical BTE usage [60,61]. Another type of BaG that gained interest is phosphate BaG (P-BaG), which enables controlled ion release. Aspects such as specific surfaces can be utilized to incorporate or graft an array of biomolecules and curative agents [62,63]. The main advantages of Si-BaG and P-BaG are their modulation of the dissolution rate and incorporation of any desired ions into the composition [64]. The scaffold obtaining technique variations regarding BaG have been explored intensely, except for electrospinning technologies [65,66].

Several studies concluded that BaG accelerates the degradation of polylactides (PLA) as well as poly-L, DL-lactide (PLDLA) [67]. Other papers concluded that composites that included BaG showed enhanced bone generation during longer periods of time and celerity in the molecular weight decrease of PLDLA when utilized in composites such as PLDLA/13-93 BaG [68]. The PLDA/13-93 BaG was also involved in the regulatory process of endothelial markers [69].

Several classifications have been established for BaG in order to see the proper domain of application and the technological flux required to achieve the specifications [70,71]. One option is the sol-gel technique, which involves the addition of a surfactant forming the desired structure of the material. The process involves a calcination step with a temperature of 700 °C, which ensures the removal of organic components obtaining the cavitated material [72].

In some applications, micrometric pore size can present a great advantage when the functionalization of the BaG needs to be realized with larger molecular weight substances [73]. The porosity of the material has been proven to be influential in processes such as remineralization [74].

## 5. Mesenchymal Stem Cells Influence in BTE

Heterogenous cells do not survive at the center of grafts that exceed thickness due to the restriction of passive transport, which has a limited distance and ensures the flux of metabolites, gases, and nutrients, thus influencing the cell capability [75,76]. Mesenchymal stem cells (MSCs) generate osteoclastogenic cytokines, the receptor activator for nuclear factor kappa B ligand (RANKL), and macrophage colony-stimulating factor (M-CSF) in physiological circumstances. MSCs co-culturing also lowered the tumorigenicity for ovarian cancer cells, but cancer-associated MSCs (CA-MSCs) determine angiogenesis and tumor development when in direct contact with tumor cells or discharging growth factors, cytokines, and exosomes [77]. The discrepancies between MSCs and CA—MSCs provoked interest and proved the CA-MSCs’ distinct properties [78]. The proliferation and diversity of cells are modulated by elements such as pH, dissolved gas, and shear stress [79,80].

Bioreactors can be used to control physicochemical factors such as pH, pressure, humidity, temperature, dissolved oxygen, carbon dioxide, and shear stress [81]. Aspects such as cellular waste removal and the nutrients flux can be controlled by the fabricated medium. Consequently, bioreactors became a desirable option for BTE applications [82,83]. The microenvironment is responsible for the stemness and lineage diversity capacities of the stem cells [84]. Another factor that drastically impacts characteristics, such as morphology and cell viability, is the shear stress-induced alignment [85]. Cell activity can be modulated by the microstructure of the utilized material [86].

Some tested parameters such as runt-related protein 2 (Runx2) and collagen type I (Col1) observed with distinct shear stress indicate that differentiation happens with celerity due to the stress increase. Runx2 represents a vital marker of osteogenesis, and Col1 represents a factor of transcription that underlies the existence of bone cells [87,88]. MSCs are required to be lead to an osteogenic phenotype that can be realized by adding growth factors; another option is their direct cultivation on bone-derived ECM, which presents osteogenic features [89,90].

MSCs therapeutic potential has been observed in several clinical uses between phases I, II, and III, many studies focusing on the engraftment obstacles and diseases regarding hematopoietic stem cells. However, some MSCs such as bone marrow (BM-MSC)-derived ones can express cytokines and thus can be utilized in cancer treatments and therapeutic payloads [77].

There are several types of MSCs sources besides BM: adipose tissue (AT) and umbilical cord (UC) are suited for cell replacement therapy. In comparison to the BM harvested MSCs, MSCs from UC and AT can be collected utilizing less invasive methods. UCs present the advantage of high proliferation and can be cultivated for a long period. AT presents similarities with processed lipoaspirate cells, resulting in a large quantity of cells generated as a by-product of cosmetic liposuction, and can grow in standard culture conditions [77].

## 6. MicroRNAs in MSCs Differentiation

**Figure 3:**
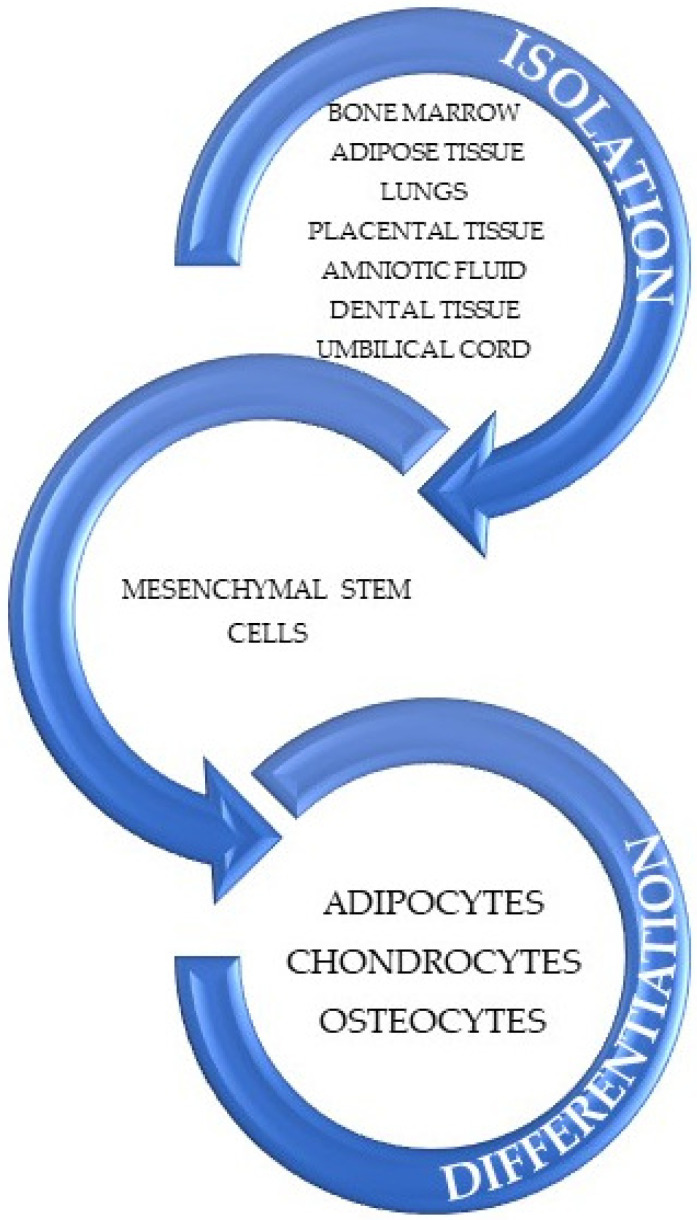
Representation of sources of MSCs and the differentiation in cell lines. Adapted from an open-access source [91].

Since the first isolation of MSCs, their ability of differentiation has been studied and evaluated as being able to act as adipocytes, chondrocytes, and osteocytes, as is displayed in Figure 3 [91,92].

Several studies have been realized on establishing what factors and signaling pathways are involved in the MSC differentiation for the purpose of BTE [93,94]. Cell differentiation has some indispensable regulators such as signals from the ECM, cytokines, and endogenous GFs [95]. Osteogenesis can also be influenced by external aspects such as mechanical forces and electromagnetic fields [96,97]. MSC osteogenic differentiation is supported by the utilized biomaterials [98,99], but the supplementation with ions enhances the osteogenic scaffold potential [100]. Cell differentiation can also be enhanced with the aid of microRNAs (miRNAs) [101], GFs [102], and biophysical stimuli [103]. Some epigenetic factors and processes that modulate the differentiation of MSCs include acetylation and methylation, non-coding RNA (ncRNAs) molecules such as miRNAs [104] and long non-coding RNAs (lncRNA) [105], and DNA methylation [106]. 

miRNAs are single-stranded ncRNAs that are responsible for regulating 30–60% of protein-coding genes. The mRNA completes the complementary stage and suffers degradation, but in the case of partial mRNA, the protein levels decrease [107,108,109,110,111,112]. One of the main tasks realized by miRNA is to bind to mRNA; apart from this gene, regulation is realized due to molecular mechanisms. The regulation can positively or negatively impact osteogenic differentiation and general transcription factors [113,114]. New approaches find a point of interest in the options that miRNAs offer in BTE [115]. They can be utilized as bioactive factors [116] and scaffolds [117] in order to generate the desired response.

There are several miRNAs involved in the generation of ROS, and thus in the oxidative stress process. Radiosensitivity can be achieved by miR-328-3p when overexpressed due to suppression of H2AX (a subtype of histone) in vitro and in vivo. NAD-dependent deacetylase sirtuin-1 (SIRT1) is capable of cell functional regulation for processes such as oxidative stress, aging, and apoptosis utilizing the deacetylation of a variety of substrates. The upregulation of SIRT1 is realized by miRNA, precisely the miR-199a, which targets the gene [118].

## 7. Osteoclast Bone Models

Osteoclasts can be defined as large, multinucleated cells which differ from monocytes and macrophages by presenting M-CSF and RANKL [119]. The co-culture techniques became popular tests for investigating bone cells such as osteoclasts, osteocytes, and osteoblasts for purposes such as bone metabolism and disease behavior [120,121,122]. Many articles have approached the co-culturing systems with osteoclasts and osteoblasts in vitro [123,124]. The main reason for the co-culture tests is to establish the interactions between cells and test different approaches in a controlled environment to generate information that can further be applied in the BTE domain [125]. The predominance of osteoblast/osteoclast co-culture studies frequency is due to the restricted availability of osteocytes. The lack of osteocytes availability resides in the difficult process of isolation for these post-mitotic cells [126,127]. There is a lack of primary osteocyte co-cultured studies with osteoclasts, with only two models reported at present, but some studies have treated the subject using murine sources for osteocytes [128]. In most cases, there are only two bone-related cell species in co-culturing techniques. The main reason is the difficulties in terms of requirements needed because of the different medium condition preparation, this being a very important step for a successful study [129]. Some results show the presence of osteoclasts, osteocytes, and osteoblasts after 21 days, even if at the beginning of the research the precursors utilized were for primary human osteoclasts and osteoblasts [125]. The setback of this study was the inability to generate data for single-cell species to investigate the matter further. Because vascularization is necessary for BTE, there has been co-culturing research involving the use of osteoclasts, osteoblasts, and endothelial cells. It has been stated in several studies that cell cultures behave in different ways in comparison with similar cells but are co-cultured, and the co-culturing technique became accepted as a common practice in biomaterial research [130]. There have been new models established that operate with triple cultures in order to obtain data on bone cellular infrastructures [131].

In the established triple cultures, all cell species showed their typical morphology and there were no obvious morphological differences between single and triple cultures. A good balance between the three cell species is a prerequisite to use those triple cultures in the future to investigate the influence of bioactive molecules, drugs, and biomaterial extracts. As expected, due to the signaling between the cells, there were detected differences between single and triple cultures on the mRNA level. Table 1 presents details regarding the techniques that are frequently used [123,128,132].

The latest model was based on generating a three-dimensional environment permitting cell–cell interactions of osteoclasts, osteoblasts, and osteocytes where the analysis can occur for all cell types as well as for single-cell species. This kind of model aims to generate a perspective on complex systems that include all the major species of bone cells. Collagen can be utilized in order to separate osteocytes from the other cells, allowing a gradually obtained result [125].

Bone cells are responsible for the development and metabolism of the bone, but there is a whole system influencing the result, which involves cytokines, GFs, receptors, transcription factors, ligands, and cell-specific enzymes [133]. The in vitro studies have a controlled medium and various options available, which also imply many variable factors that need to be addressed. Thus, the studies have many challenges to overcome. Existent models have studied vasculogenesis, bone marrow, and tumor models [134]. The models also went in a direction similar to microfluidics; approaches such as bone-on-a-chip and organ-on-a-chip lead towards a system that facilitates the investigation of osteoclasts or osteoblasts responding to osteocyte-prepared medium [135,136]. However, since microfluidics work with a limited number of samples, the popular choice remains a simpler co-culturing model strongly impacted by the collagen gel, which allows obtaining a 3D environment [137,138].

Materials such as copper are used due to the great properties they possess, such as angiogenesis and osteogenesis enhancement, as well as antimicrobial effects [139,140]. This material is used to generate enhanced BaG, titanium implants, cement, and ceramics [141,142]. The osteoclasts are influenced by the material due to the potential oxidative stress exerted at different concentrations [138].

There are four main categories of 3D co-culturing models: hydrogels, cell aggregation, scaffolds, and dynamic models. Some of them can be divided once more into subcategories, thus ensuring diversity and options for several new applications. For hydrogels we have the source, which can be natural or synthetic, the first being a network composed out of naturally originated monomers and the latter being synthetic. Both present different advantages and disadvantages according to the source and profile, even though diffusion plays a large role in the success of both. The cell aggregation can be classified with the technological flux, one being obtained with hanging drop plates and the other being generated with 3D Petri dishes. The first one generates 3D spheroid aggregates and the second technique molds agarose in order to obtain defined shapes by cell agglomeration. The scaffolds can also be differentiated with the source of the material dividing it into synthetic and natural 3D structures [123].

There are several new theories that will increase the complexity of the existing 3D models. Three-dimensional models may replicate the in vivo conditions while using human cells in order to obtain valid information regarding the hypothesis obtained with the animal trials which require further analysis. Furthermore, techniques such as perfusion and biomechanical mimetism have created room for studies that generate similar conditions to the in vivo environment, thus becoming a preferred model for researchers. Also, they ensure a high compatibility rate and diversity, alongside good chemical properties [123].

For instance, Zhang and colleagues [143] proposed a complex 3D structure-based co-culture platform that mimics the Haversian bone, with osteogenic cells angiogenic/neurogenic cells distributed at a specific location for active bone tissue engineering. The authors have used digital laser processing-based 3D printing technology to ensure the precision of their scaffolds, allowing the creation of custom-designed structures. The as-described system displayed significantly improved osteogenic and angiogenic effects as compared with the unicellular delivery system both in vitro and in vivo, holding great promise for tissue regeneration.

Moreover, recent progress has been reported in moving from preclinical 3D models to clinical models [144]. For example, Pauli et al. [145] have described the development of a precision cancer care platform integrating whole-exosome sequencing with a living biobank that enables high-throughput drug screens on patient-derived tumor organoids. The scientists investigated 56 tumor-derived organoid cultures and 19 patient-derived xenograft models, including bone models, from 769 patients enrolled in an Institutional Review Board-approved clinical trial. Their tremendous effort may serve as a basis for discovering novel personalized therapeutic options, especially for patients where standard clinical options have been exhausted.

Several other perspectives in the field of 3D bone culturing models include automating cell seeding procedures towards attaining a safe and standardized production of engineered tissue constructs, using nondestructive live-monitoring techniques to obtain unique insights into cellular interactions, and moving from single culture to multiorgan models in order to understand the molecular communications between the bone tissue model and other tissues/organs [146]. 

## 8. Oxidative Stress Influence in BTE

**Figure 4:**
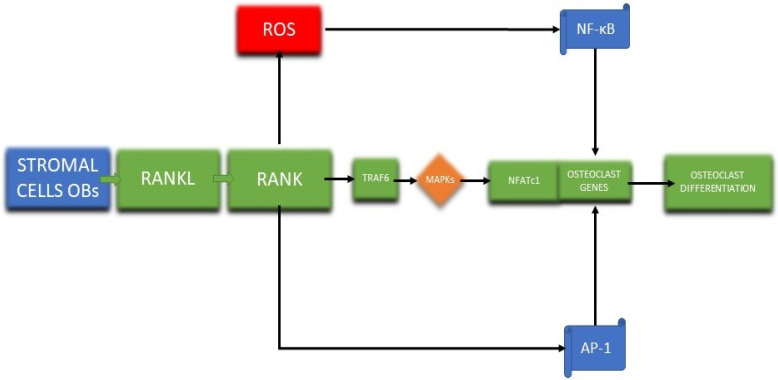
The signaling pathway for osteoclasts differentiation (adapted from an open-access source) [148].

Osteoblasts and osteoclasts are vital in bone remodeling; thus, they have been investigated to better comprehend the bone regeneration mechanism [147,148]. After the discovery of osteoprotegerin (OPG), RANKL, and RANK, a pathway was formed which supplies data for the osteoblast regulation of osteoclasts utilizing bone matrix interaction, paracrine factors, and cell–cell contact [123,149]. Osteoclasts differentiation (Figure 4) starts with the attachment of RANKL to the RANK receptor with the aid of a receptor-associated factor (TRAF6) which activates a wide range of mitogen-activated protein kinases (MAPKs) that trigger the nuclear factor of activated T cells 1 (NFATc1), leading to osteoclasts differentiation [148].

Reactive oxygen species (ROS) are molecules and free radicals (e.g., superoxide anion, hydrogen peroxide, hydroxyl radical) mainly resulting as byproducts of leaked electrons from the mitochondrial electron transport chain during aerobic respiration [118]. ROS are necessary for regulating cellular processes, including proliferation, survival, metabolism, apoptosis, and differentiation. ROS are moieties of interest in bone regeneration as they can be used for dual purposes. Their beneficial aspect resides in the ability to act as an intracellular marking agent, being essential for the transmission of cell signals [148].

However, ROS can produce cellular imbalance in reduction–oxidation reactions when their level increases due to age or inflammatory states, leading to oxidative stress. Oxidative stress modulates fundamental cellular physiological responses via signal transduction, transcription factors, and ncRNAs, promoting nuclear and mitochondrial DNA damage and initiating DNA repair pathways [118,148]. Continued oxidative stress was demonstrated to generate diseases, leading to bone destruction and cellular death [149]. 

ROS are also vital components in osteoclasts’ regulation of differentiation [150]. It had been assumed that the extreme production of osteoclasts induced by local inflammation could be prevented by limiting the excessive production of intracellular ROS. The impact of ROS in the relationship of osteoclasts and osteoblasts was studied with the help of co-cultured models [151]. In particular, maintaining bone homeostasis is critical in preserving an optimum balance between formation and resorption, influencing bone mass and strength. Specifically, these properties begin to reduce with aging, with an enhanced osteoclast activity and a decreased osteoblast activity [148].

Cellular senescence results in a response to persistent stress; it is characterized by a stable cell-cycle arrest, the expression of senescence-associated β-galactosidase (SA-β-gal), the increased expression of the cell cycle inhibitor p16^Ink4a^, the overexpression of the cyclin-dependent kinase (CDK) inhibitor, senescence-associated secretory phenotype (SASP), telomere shortening, and a persistent DNA damage response (DDR) [118,149]. This is relevant in the context of oxidative stress as ROS provoke cell senescence by stimulating the DDR pathway to stabilize p53 and promote CDK inhibitor gene expression. Particularly, p53 represents a major regulator of cellular response to oxidative stress. On the one hand, it can decrease ROS levels and repair DNA damage in cells; on the other hand, p53 can increase ROS production and promote the apoptosis or senescence of the cells [118].

Moreover, several conditions can occur due to the patient’s age, such as postmenopausal osteoporosis, diabetes, cirrhosis, cancer, and neurogenerative disease, which can be delayed with antioxidants related to chemo-preventive and curative therapies such as glutathione [152,153,154,155]. The redox indicators that are usually analyzed are reduced glutathione (GSH) and oxidized glutathione (GSSG) and their balance. In addition, some studies noted the increase of osteoclasts differentiation in the presence of GSH [156].

One of the popular antioxidants used for clinical studies and cell cultures is N-acetyl cysteine (NAC) [157,158]. Studies that involve NAC treatments concluded a reduction in cellular processes, and ROS have some involvement in the matter. NAC and ascorbate have beneficial effects in reducing stimulus for the loss of bone mass, osteoblast apoptosis, oxidative stress, and osteoclastogenesis after gonadectomy [159,160]. NAC has also been utilized in the analysis of mitochondrial ROS and physiological involvement [161]. Important results have been also obtained by the oral administration of Ewha-18278 (a pyrazole derivative). It was reported that Ewha-18278 protected ovariectomy-induced osteoporosis in mice by NADPH oxidase (NOX) inhibition and ROS suppression. This anti-osteoporotic agent aided in the recovery of bone parameters, leading to improved bone strength and an increased number of osteoblasts [148]. Another beneficial effect was registered from vitamin K managing to reduce oxidative stress and ROS production. Specifically, vitamin K was observed to protect cells from H_2_O_2_-induced changes in protein expression, being able to support bone tissue formation, remodeling, and mineralization [162,163].

Phosphoinositide 3-kinase (PI3K)/protein kinase B (AKT) represents a signaling pathway that regulates cells’ proliferation, survival, and death and the osteoblastic and osteoclastic functions altering the formation, differentiation, and apoptosis. The deficiency of AKT2 translates into a decrease in RUNX2 expression. Thus, the AKT pathway promotes RUNX2 gene expression [164]. Osteogenic differentiation is promoted by the regulation of PI3K/AKT and RUNX2, modulating the RUNX2 activity [165].

Table 2 comprises several clinical studies that are being realized, researched, or revisited. The trials reflect the focus growth that the domain is gaining due to the continuous discoveries in the field. In time, the data and the enhancement of cell isolation will allow more clinical trials to take place. However, the stages in which some studies reside are incipient and still focus on side effects and dosage for emerging solutions.

## 9. Discussion

Bone regeneration requires the top performance of several components such as efficient analysis, materials that possess special properties, suitable GFs, and molecules to ensure a successful overall process. 

Oxidative stress plays a vital part in bone regeneration as it is known to alter the process of bone remodeling. Through the unbalance it creates between osteoblasts and osteoclasts activity, persistent exposure to high levels of oxidative stress may further result in cellular senescence, bone diseases, and skeletal system disorders. Thus, special consideration must be given to maintaining oxidative stress at optimum levels. Particularly, the destructive potential of ROS can be reduced by the administration of antioxidant agents that can restore bone parameters, supporting bone tissue formation, remodeling, and mineralization.

Nonetheless, ROS production can also be considered relevant as a therapeutic target under certain conditions, thus being a beneficial aspect if tackled properly. More exactly, ROS can act as an intracellular marking agent, being involved in the transmission of cell signals. Hence, its detection and targeting can contribute to a better understanding of cellular interactions and approaching pathophysiological conditions in a more specific manner. Therefore, oxidative stress needs to be further regulated and researched in order to benefit from good results in the field. 

Bone regeneration can be enhanced by use of specifically engineered scaffolds. Materials such as BaG and hydroxyapatite remain good performers among others which possess advantageous properties, such as a low cytotoxicity and good biocompatibility. This provides a strong basis for the functionalization and regeneration of the tissue.

Culturing systems are also important tools in analyzing the mechanisms of bone regeneration, bone homeostasis, and ROS generation and effectively evaluating the various cells’ response to emerging BTE strategies. In particular, the 3D techniques generate new data and legitimization to animal studies that were never performed on humans before. Also, the mimetism of the in vivo domain makes it a good perspective to take into consideration when it is time to start clinical studies.

**Figure 5:**
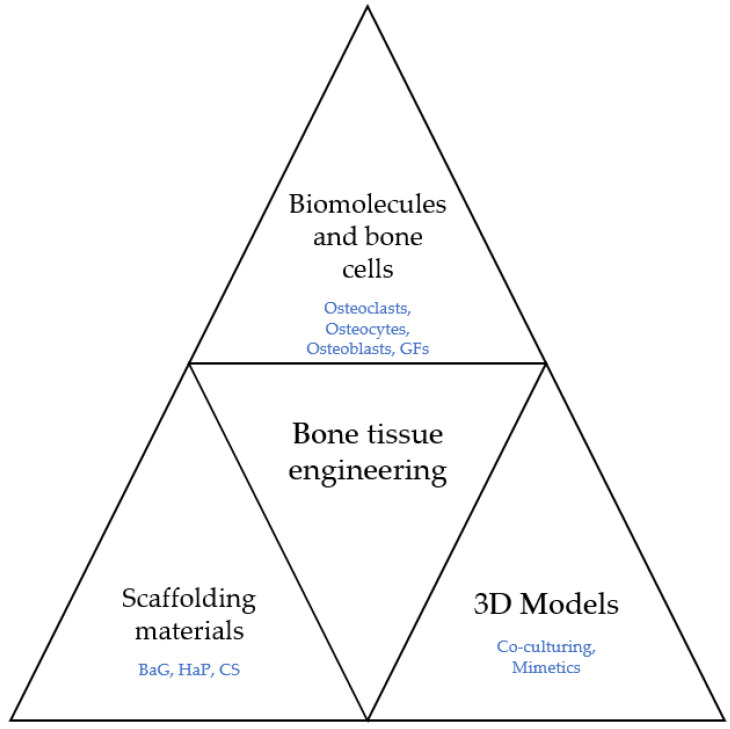
The fields involved in BTE.

Growth factors such as VEGF modulate the process of angiogenesis and osteogenesis. Hence, they represent valuable assets to any strategy when used properly. Figure 5 displays the main fields that take part in BTE.

To summarize the discussion on bone regeneration and oxidative stress, Table 3 synthesizes the roles and effects of the various described cells, growth factors, biomolecules, signal pathways, and bone engineering scaffolds.

## 10. Conclusions and Future Perspectives

Bone regeneration depends on several other domains to generate the best results, with many discoveries and breakthroughs owed to interdisciplinary research studies. Bioactive materials have been researched independently and used under the form of composites to obtain synergistic results monitored through in vitro and in vivo tests. The materials response leads to the discussion of functionalization with biomolecules and growth factors to accelerate the healing process with the full potential of cell growth. Aspects and particularities such as tensile strength, pore dimension, and composition are frequently modulated to obtain a data flux that can help decide the desired path to be followed. 

As bone cells are scarce, not much research is available in clinical settings. Thus, studies mostly concern the employment of culture models for investigating bone cell interactions, bone metabolism, and disease behavior. In this respect, particular attention has been drawn to coculturing osteoclasts, osteocytes, and osteoblasts into biomimetic platforms. The co-cultured models have also sparked the idea of microfluidic attempts to generate bone-on-a-chip and ensure reliable data, but lack the ability to obtain a wide array of samples to date. Interesting results are also expected from the future development of multiorgan models that would help comprehend the importance of bone homeostasis in relation to other tissues and organs.

Considering every aspect that the domain provides, the existing solutions are continuously enhanced with the availability of new data streams. Oxidative stress and its associated diseases are being analyzed, and new antioxidants emerge as promising solutions. The beneficial aspects of ROS and the damaging concentrations of the molecules are being tested to have the greatest benefits from the data obtained. 

To conclude, multiple bone scaffolding materials and functionalization molecules are emerging from recent studies and might soon start being utilized for obtaining a better clinical experience. Thus, we can presume that developing interdisciplinary studies lead to the emergence of new technologies for tissue engineering, oxidative stress control, osteogenesis promotion, and bone disease prevention.

## Figures and Tables

**Table 1:** Advantages and limitations of the culturing techniques. Created based on information from [123,128].

Culturing Type	Particularities	Observations	Advantages	Limitations
Triple culture of osteoblasts, osteoclasts, and osteocytes	• There are two techniques for seeding: one done individually called patterned seeding and the other being called mixed seeding	• Patterned seeding resulted in a better individual analysis • Mixed seeding generated better direct cell-cell contact	• Realistic model • Allows the study of the interactions of the cells • No major morphological differences • The ratio of cells to substance increases	• The models are not cultivated on resorbable membranes • Different serum concentrations are required for differentiation • Requires more substances and space
Co-cultured approaches with two different species of bone cells	• The test was realized with a porous membrane situated between the two cultures promoting the single cell type analysis	• The osteoclast gels present poor strength in comparison with the single culture underlining the degrading osteoblastic enzyme outcome	• Analytical aim of crosstalking cultures • Cost-effective	• The resorption capacities of the cultures are yet to be determined • Fewer variation opportunities resulting in isolated outcomes

**Table 2:** Examples of clinical studies in the domain of BTE.

Clinical Trials • gov• Identifier	Official Title	Purpose of the Study	Data Availability
NCT03652753	Pilon Fracture With Intra-articular Injection of N-Acetylcysteine (Pilon NAC)	Analyzation of the outcomes obtained from the amino acid NAC on cartilage cells in cases of intra-articular fracture of the ankle joint.	Estimated primary completion date: January 2023 Estimated study completion date: January 2024
NCT03024008	Enhancement of Bone Regeneration and Healing in the Extremities by the Use of Autologous BonoFill-II	Evaluation of the safety of an autologous bone-regenerating graft for the reconstruction of deficient bone.	Estimated primary completion date: June 2022 Estimated study completion date: September 2022
NCT04498715	Trochanteric Femur Fracture Operated With Dynamic Hip Screw System (DHS) Augmented With a Biphasic Apatite Sulphate Combined With Systemic or Local Bisphosphonate	Investigating the bone regeneration processes for a metal device utilizing bone substitute cement and bisphosphonate.	Estimated primary completion date: February 2021 Estimated study completion date: August 2021
NCT02171104	MT2013-31: Allo HCT for Metabolic Disorders and Severe Osteopetrosis	The assessment of the capacity to generate donor hematopoietic engraftment without growth in transplant-related mortality for patients with severe osteopetrosis and inherited metabolic disorders.	Estimated primary completion date: September 2021 Estimated study completion date: December 2021
NCT04875767	Cartilage Repair Using a Hyaluronic Acid-Based Scaffold With Bone Marrow Aspirate Compared With Microfracture for Focal Articular Cartilage Damage of the Hip (CHASE)	Investigating the results of the procedure for a 24-month timespan post-surgery in order to determine if any complications will occur.	First posted: 6 May 2021 Estimated study completion date: 31 December 2026

**Table 3:** Overview of relevant elements for bone regeneration and oxidative stress.

Key Elements		Roles/Effects in Bone Regeneration and Oxidative Stress	Refs.
Category	Representatives		
Cells	Osteoblasts	Can promote calcium salts deposition in the bone matrix, leading to bone reconstruction Their increased production is associated with improved fracture healing time Secrete OPG	[105,166]
	Osteoclasts	Responsible for bone resorption Involved in bone remodeling, which is an essential process for regeneration of bone defects Express M-CSF and RANKL Their increased cellular activity has been associated with bone diseases, such as osteoporosis, rheumatoid arthritis, and osteoarthritis	[119,148,166]
	Osteocytes	Regulate osteoclast and osteoblast activity Optimally used and stimulated osteocytes lead to improved bone regeneration Can enhance osteogenesis of stem cells Feedback from osteocytes limits the size of the bone-forming unit by the secretion of sclerostin	[150,167]
	MSCs	May differentiate into osteocytes Generate osteoclastogenic cytokines, RANKL, and M-CSF Inducing MSC osteogenesis promotes bone formation and bone regeneration	[77,91,105]
Growth factors, biomolecules, and signal pathways	VEGF	Regulates osteoclast activity Modulates angiogenesis and osteogenesis Facilitates MSCs homing and differentiation Elevates intracellular ROS level	[36,37,166,168,169]
	FGF	Active role in bone repair process Modulates osteoblasts differentiation Enhances bone regeneration in bone defects and clinical fractures	[13,170]
	BMP	Regulator of cartilage and bone formation Modulates osteoblasts differentiation Facilitates MSCs homing and differentiation	[13,105,168]
	Shh	Upregulates BMPs Modulates osteoblasts differentiation Stimulates a cascade of downstream genes involved in bone development Enhances regeneration in bone defects	[13,171]
	M-CSF	Modulates osteoclasts differentiation Role in recruiting stem cells to the fracture site Impacts hard callus formation	[13,172]
	RANKL	Modulates osteoclasts differentiation Can enhance osteoclastogenesis and improve bone remodeling when added to biomaterials Can induce ROS formation	[13,150,166]
	OPG	Antagonist receptor for RANKL Decreases osteoclast formation and bone resorption activity	[166,173]
	NOX	One of the main sources of ROS NOX2-derived O_2_^−^ enhances RANKL-induced NFATc1 expression in osteoclast signalling; NOX2 inhibition exerts protective effects and may prevent bone loss NOX4 contribues to osteoclastogenesis and bone homeostasis; NOX4 is involved in bone loss, representing a potential therapeutic target for osteoporosis treatment	[148]
	P53	Regulates cell cycle, apoptosis, growth, and metabolism of target genes Master regulator in the cellular response to oxidative stress Reduces intracellular ROS levels by promoting antioxidant reactions	[118]
	miRNA	Modulates osteogenic differentiation and general transcription factors Can regulate processes such as oxidative stress, aging, and apoptosis Involved in the generation of ROS Several miRNAs are involved in cellular senescence	[101,118]
Scaffolds	Hydrogels	Delivery and controlled release of growth factors that aid neovascularization Enhance proliferation of HUVEC when incorporated with these cells Support co-culturing of bone cells	[21,34,123]
	Bioceramics	Their microstructure promotes ossification and vascularization growth Well-defined pore architecture improves cell seeding efficiency, cell viability, migration, proliferation, and differentiation, enhancing bone regeneration Can be functionalized with growth factors that aid in osteogenesis and angiogenesis	[25,34,35,49]
	Bioactive glass	Enhances bone regeneration during longer periods of time Involved in the regulatory process of endothelial markers Material porosity influences remineralization	[68,69,74]

Abbreviations: MSCs—mesenchymal stem cells; VEGF—vascular endothelial growth factor; FGF—fibroblast growth factor; BMP—bone morphogenic protein; Shh—sonic hedgehog; M-CSF—macrophage colony-stimulating factor; RANKL—receptor activator of nuclear factor kappa-B ligand; OPG—osteoprotegerin; NOX—NADPH oxidase; NFATc1—nuclear factor of activated T cells 1; HUVEC—human umbilical vein endothelial cells.
